# Association between duration of dysphagia and dysphonia with insomnia: results from the National Health Interview Survey

**DOI:** 10.3389/fneur.2026.1796030

**Published:** 2026-05-08

**Authors:** Kai Zhou, Wen Zhao, Jiehua Gan, Guomin Xie, Xiping Wu, Zhongyue Lv

**Affiliations:** Department of Neurology, The Affiliated Lihuili Hospital of Ningbo University, Ningbo, Zhejiang, China

**Keywords:** dysphagia, dysphonia, insomnia, NHIS, risk factor

## Abstract

**Background:**

Insomnia markedly impairs health and quality of life. This study used the 2022 National Health Interview Survey (NHIS) data to explore the relationships among dysphagia duration, dysphonia duration, and insomnia.

**Methods:**

With the 2022 NHIS data, participants were stratified into insomnia and non-insomnia groups. Chi-square tests assessed group differences in dysphagia/dysphonia duration and covariates. Weighted multivariate logistic regression evaluated associations between dysphagia duration, dysphonia duration, and insomnia, and risk stratification was used to validate correlation stability. Feature importance analysis identified the contribution of each feature to the model.

**Results:**

In this study, 1,617 subjects were recruited when dysphagia duration served as the exposure factor (548 with insomnia and 1,069 without), and 3,130 subjects were included when dysphonia duration served as the exposure factor (844 with insomnia and 2,286 without). Baseline characteristics showed that dysphagia and dysphonia durations significantly affected insomnia outcomes (*p* < 0.05). Weighted multivariate regression confirmed these as independent risk factors (odds ratio (OR) > 1, *p* < 0.05 in all models). Notably, this effect remained largely unchanged after adjusting for other covariates. Finally, dysphagia and dysphonia durations ranked among the top in the feature importance results.

**Conclusion:**

This study identified dysphagia and dysphonia durations as independent risk factors for insomnia, with their effects persisting after adjusting for potential covariates. These findings highlight the importance of considering swallowing and voice disorders in insomnia prevention strategies.

## 1 Introduction

Insomnia is the most common sleep disorder, characterized by recurrent difficulties in falling asleep, sleep maintenance disorders (e.g., awakening ≥2 times per night), and early-morning awakening despite having adequate opportunity to rest (1–3). These symptoms lead to reduced total sleep time and impaired sleep quality (4, 5). Epidemiological evidence suggests that roughly 10% of adults meet the diagnostic criteria for insomnia, while an additional 20% experience occasional symptoms (6). This sleep disorder is more commonly observed among women, older adults, and patients with cancer (7–9). Physiological, psychological, behavioral, and socioenvironmental influences contribute to insomnia (10, 11). Current primary treatment approaches for insomnia include pharmacotherapy and cognitive behavioral therapy for insomnia (CBT-I). Among these, CBT-I is widely recognized as the most effective and comprehensive nonpharmacological intervention, integrating cognitive therapy, behavioral modification, and sleep hygiene education (12). Despite evidence that CBT-I offers a much more favorable benefit–risk profile than pharmacological treatments, some individuals with insomnia do not achieve full benefit from this intervention (13, 14). If left untreated, insomnia can progress to a chronic state, potentially resulting in cognitive decline, social dysfunction, and various physiopsychological comorbidities, thus markedly impairing quality of life (15). Therefore, identifying its risk factors and implementing timely interventions are essential for reducing insomnia incidence, preventing disease progression, and alleviating the associated societal and familial burden.

Dysphagia is commonly categorized as oropharyngeal dysphagia (OD) and esophageal dysphagia (ED) (16, 17). OD, often associated with chronic neurological disorders such as stroke, Parkinson’s disease, and dementia, presents with swallowing difficulties, coughing, choking, or aspiration, whereas ED typically manifests as a sensation of food sticking after swallowing (16–20). OD has an estimated prevalence of 43.8% globally, with the highest prevalence in Africa (64.2%) (21). Potential mechanisms linking dysphagia to insomnia may involve nocturnal airway compromise, recurrent coughing or choking episodes, and an increased risk of aspiration, all of which can disrupt sleep continuity (22). In addition, dysphagia is frequently associated with neurological disorders, which may affect sleep regulation (23). Psychological distress, including swallowing difficulty–related anxiety, may further contribute to impaired sleep initiation (24). For example, a case report described a 71-year-old woman developing insomnia following dysphagia onset (25); moreover, epidemiological studies among older Japanese adults have reported that dysphagia risk correlates with sleep quality (26). Dysphonia arises from impaired voice production, resulting in abnormalities during speaking or vocalization (27). Dysphonia can impair communication and social relationships and is also closely linked to quality of life. Insufficient and excessive sleep durations are markedly associated with an increased risk of developing dysphonia (28). However, existing studies are largely limited to small samples, specific populations, or cross-sectional observations without a detailed assessment of symptom duration. In particular, the impact of the symptom duration of dysphagia and dysphonia on insomnia remains undercharacterized. Understanding these associations may provide important insights into early identification and targeted interventions for insomnia.

The National Health Interview Survey (NHIS) provides nationally representative data with standardized data collection, enabling the investigation of associations between health conditions at the population level (29, 30). Using NHIS data, one study investigated trends in sleep difficulties among the U.S. population between 2013 and 2017 (31). Another analysis based on the same database revealed that individuals born in the 1950s and 1960s were significantly more likely to report shorter sleep durations compared to those from earlier birth cohorts (32). Collectively, these lines of evidence indicate that sleep problems vary markedly across time periods and among specific populations. However, the current exploration of the association between specific physical symptoms (such as dysphagia and voice disorders) and insomnia remains insufficient. Therefore, this study aims to utilize NHIS data to conduct an in-depth investigation into the relationships among dysphagia, dysphonia, and insomnia in order to address the gaps in existing research. We used the 2022 NHIS data to explore the association between dysphagia and dysphonia duration and the risk of insomnia in a nationally representative population.

## 2 Materials and methods

### 2.1 Data collection

**Figure 1:**
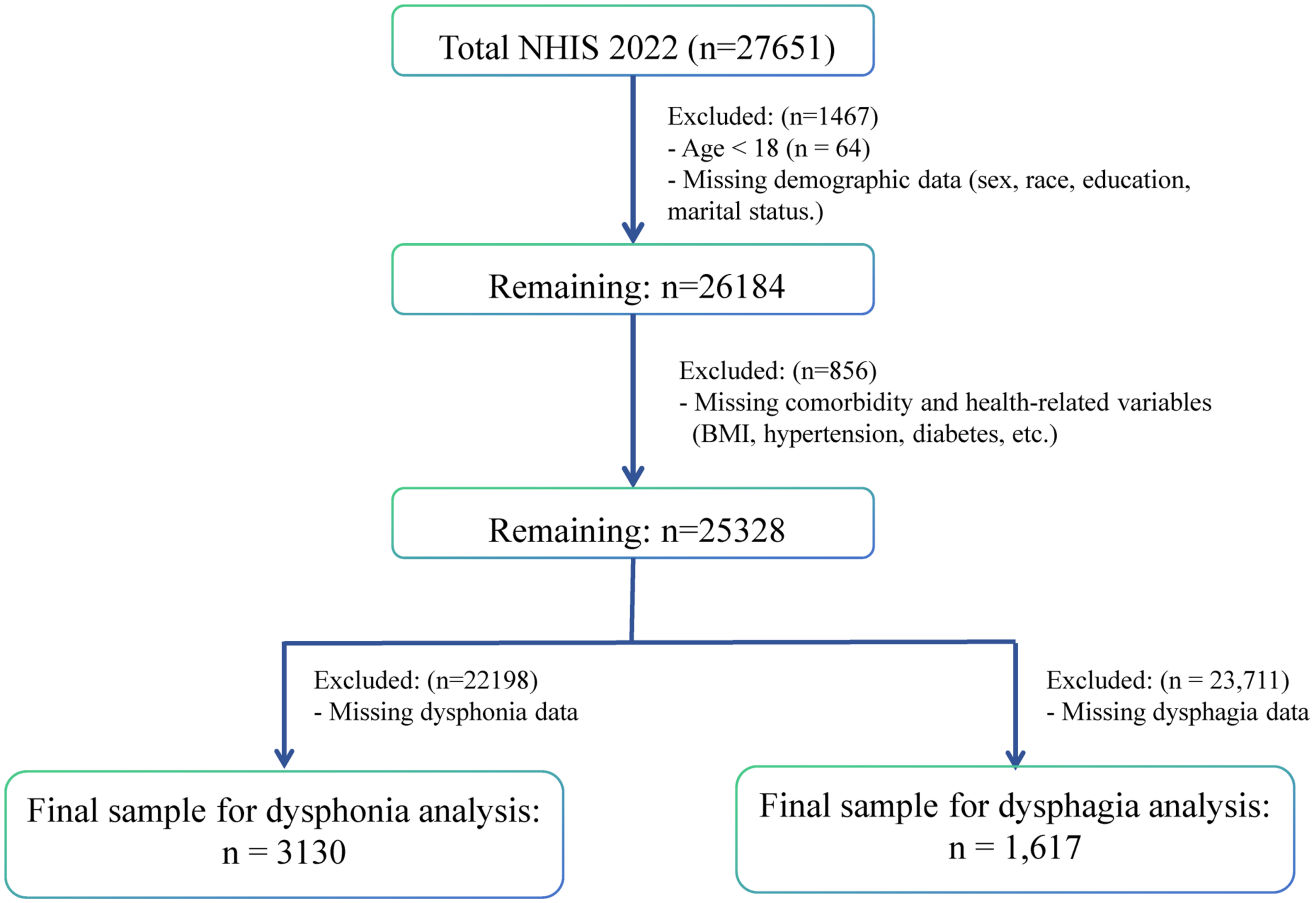
Flow diagram of participant selection from the NHIS 2022 dataset. Following stepwise exclusion of participants with missing data, separate analysis cohorts were established for dysphagia (*n* = 1,617) and dysphonia (*n* = 3,130) analyses.

**Figure 2:**
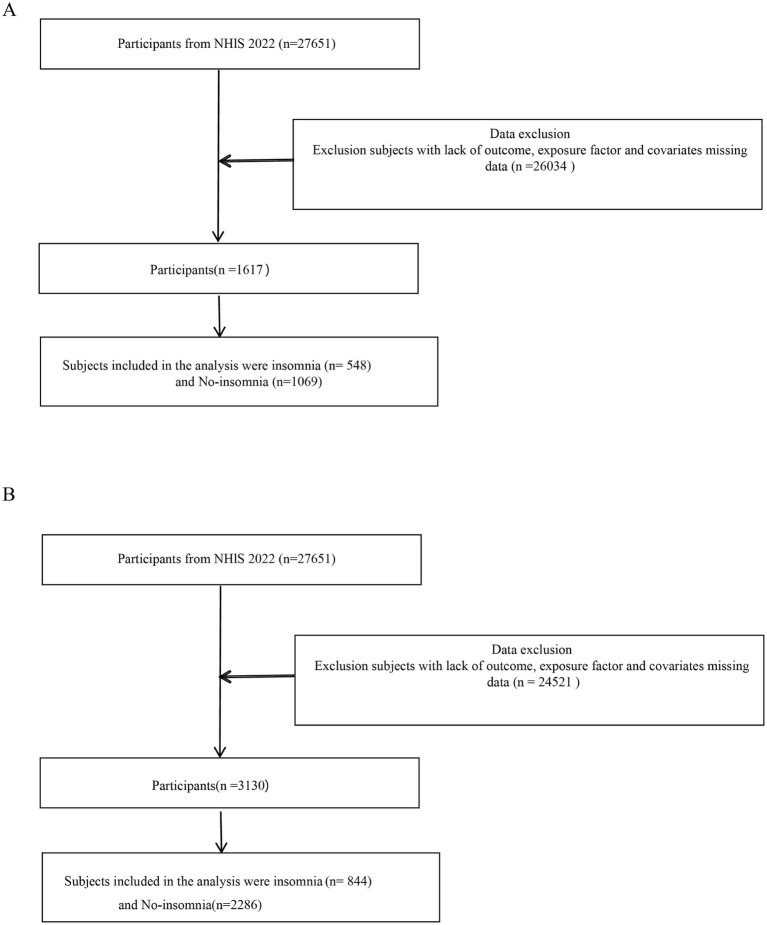
Flowchart of inclusion and exclusion of participants from the 2022 NHIS database. **(A)** Flowchart for the analysis of dysphagia duration as the exposure. **(B)** Flowchart for the analysis of dysphonia duration as the exposure.

In this study, the Sample Adult Interview option was chosen from the NHIS database,^1^ and the data of all adult subjects in 2022 (all of whom provided written informed consent forms) were collected. Exclusion criteria included subjects with missing outcomes, exposure, or covariate data. Figure 1 presents a detailed flowchart of the participant selection process. When dysphagia duration served as the exposure factor, 1,617 subjects were recruited (548 with insomnia and 1,069 without). When dysphonia duration served as the exposure factor, 3,130 subjects were recruited (844 with insomnia and 2,286 without) (Figures 2A,B).

### 2.2 Definition of outcome and exposure factors

Insomnia was assessed using the NHIS item PHQ83_A, which asks: “How often have you had trouble sleeping in the past 2 weeks?” Responses were recorded on a 4-point scale: (1) not at all, (2) several days, (3) more than half the days, and (4) nearly every day. While this self-reported measure is not a clinical diagnosis (e.g., DSM-5 or PSG), such validated items are standard, widely accepted surrogates for evaluating sleep quality in large-scale epidemiological studies (33). Following previous studies, responses were dichotomized into a binary variable: participants reporting “more than half the days” or “nearly every day” were classified as having insomnia, while those reporting “not at all” or “several days” were classified as not having insomnia. This cutoff provides good sensitivity and specificity for identifying clinically relevant sleep disturbances (34).

Definition of Exposure Factors: Enter the NHIS database, select the data for the year 2022, choose Sample Adult Interview, and search for “VSLSWDYS_A.” Then, obtain the question “During the past 12 months, about how many days, weeks, or months did you have problems or difficulty with swallowing?” Classify the relevant answers according to the dysphagia duration in them, which are 1–6 days, 7–13 days, 14–29 days, 30–89 days, 90–179 days, and ≥180 days. Similarly, search for “VSLVDYS_A” to get the question “During the past 12 months, about how many days, weeks, or months did you have problems or difficulty with voice?” Divide the relevant answers into six categories according to the dysphonia duration in them: 1–6 days, 7–13 days, 14–29 days, 30–89 days, 90–179 days, and ≥180 days.

### 2.3 Definition of covariates

Inclusion of covariates related to insomnia: Sociodemographic factors include Gender, Age, Race, Region, Education, Marital_Status, Employment, Income, and Smoke. Health status factors include Health, Mental, Disability, and Body Mass Index (BMI). Specifically, “Health” was defined based on self-reported general health status (PHSTAT_A), “Mental” was derived from mental health treatment status (MHTHRPY_A), “Disability” was defined using functional limitation status (DISAB3_A), and BMI was categorized based on BMICAT_A. Related diseases include Heart_attack, Cancer, Asthma, Hypertension, High_cholesterol, Arthritis, Coronary_heart_disease, Stroke, and Prediabetes. Healthcare data includes Health_Insurance (Supplementary Table 1). These covariates were included as potential confounders based on prior literature, given their established associations with insomnia.

### 2.4 Model establishment

Three weighted multivariate logistic regression models with different covariate adjustments were constructed to examine the associations among dysphagia duration, dysphonia duration, and insomnia. Model definitions: Model 1—An unadjusted model; Model 2—Adjusted for Gender, Age, and Race; Model 3—Further adjusted on the basis of Model 2 by including Region, Education, Marital_Status, Employment, Income, Smoke, Health, Mental, Disability, BMI, Heart_attack, Cancer, Asthma, Hypertension, High_cholesterol, Arthritis, Coronary_heart_disease, Stroke, Prediabetes, and Health_Insurance.

### 2.5 Statistical analysis

The study data were statistically analyzed using R software (v 4.2.2), with a fixed random seed (seed = 123) to ensure reproducibility. The chi-square test (*p* < 0.05) was applied to categorical variables using the “tableone” package (v 0.13.2) (35). The chi-square test was employed to compare baseline characteristics between the insomnia and non-insomnia groups, allowing for the initial identification of categorical variables significantly associated with the outcome. Statistical analyses were performed using the “glmnet” package (v 4.1.8).

Multivariate logistic regression was used as the primary analytical framework to estimate odds ratios (ORs) and 95% confidence intervals (CIs), prioritizing the interpretability of independent associations between dysphagia/dysphonia duration and insomnia (36). This method was selected to control for potential confounding variables, thereby enabling the estimation of independent associations (ORs) between dysphagia/dysphonia duration and insomnia, which is essential for etiological inference. OR values above 1 denote risk factors, values below 1 denote protective factors, and a value of 1 denotes no association (*p* < 0.05). Unlike data-driven approaches such as LASSO or machine learning (e.g., XGBoost), which are optimized for high-dimensional feature selection or predictive performance, our approach focuses on robust epidemiological association and etiological explanation.

To assess the robustness of the fully adjusted model (Model 3), repeated 10-fold cross-validation (*K* = 10, repeats = 5) was performed. The model performance was evaluated using the area under the receiver operating characteristic curve (AUC) and accuracy across resampling iterations. To further examine the stability of the associations, regression coefficients, ORs, and *p*-values for the primary exposure variables were extracted from each resampled model. The consistency of effect direction (sign consistency) and the proportion of statistically significant results (*p* < 0.05) were calculated. In addition, variable importance was assessed based on the absolute Wald Z statistics, and the stability of variable ranking across resampling iterations was evaluated.

The “forestplot” package (v 3.1.5) (37) was used to draw a forest plot to display the results. Finally, the “caret” package (v 6.0.94) (38) was employed to calculate the importance scores of each feature and rank them to evaluate the contribution of each feature to the model and its impact on insomnia. A *p*-value of less than 0.05 was considered statistically significant. Feature importance analysis was conducted to quantitatively rank the relative contribution of each variable to the model. This complements the regression analysis by identifying the most influential predictors of insomnia risk from a holistic perspective.

## 3 Results

### 3.1 Statistical analysis of baseline characteristics of the study subjects

To assess differences in insomnia across exposure factors, the subjects were divided into insomnia and non-insomnia groups based on the presence or absence of insomnia. The chi-square test assessed differences in exposure factors and covariates between the two groups (*p* < 0.05). Table 1 shows that dysphagia duration significantly affected insomnia outcome (*p* < 0.05), as well as other potential influencing factors, such as Age, Education, Marital_Status, Employment, Income, Smoke, Health, Mental, Disability, BMI, Heart_attack, Prediabetes, Hypertension, Arthritis, Coronary_heart_disease, Stroke, and Asthma (*p* < 0.05) (Table 1). Furthermore, the dysphonia duration markedly affected insomnia outcomes (*p* < 0.05), as well as 18 covariates including Age, Education, Marital_Status, Employment, Income, Smoke, Health, Mental, Disability, BMI, Heart_attack, Prediabetes, Hypertension, High_cholesterol, Arthritis, Coronary_heart_disease, Stroke, and Asthma (*p* < 0.05) (Table 2).

**Table 1:** Baseline characteristics of participants with dysphagia by insomnia status.

Variables	Level	Non-insomnia (*n* = 1,069)	Insomnia (*n* = 548)	*p-*value
Age	≥65	424 (39.7%)	202 (36.9%)	0.005
	18–29	134 (12.5%)	56 (10.2%)	
	30–39	149 (13.9%)	60 (10.9%)	
	40–49	134 (12.5%)	69 (12.6%)	
	50–64	228 (21.3%)	161 (29.4%)	
Gender	Female	612 (57.2%)	337 (61.5%)	0.112
	Male	457 (42.8%)	211 (38.5%)	
Race	Hispanic (all other groups)	50 (4.7%)	23 (4.2%)	0.248
	Hispanic (Mexican/Mexican American)	81 (7.6%)	30 (5.5%)	
	Not hispanic	938 (87.7%)	495 (90.3%)	
Education	High school grad	249 (23.3%)	150 (27.4%)	0.001
	Less than high school grad	81 (7.6%)	65 (11.9%)	
	More than high school grad	739 (69.1%)	333 (60.8%)	
Marital status	Living with partner	64 (6.0%)	45 (8.2%)	<0.001
	Married	480 (44.9%)	180 (32.8%)	
	Neither	525 (49.1%)	323 (58.9%)	
Employment	No	559 (52.3%)	354 (64.6%)	<0.001
	Yes	510 (47.7%)	194 (35.4%)	
Income	Middle and high income	723 (67.6%)	307 (56.0%)	<0.001
	Nearly poor	205 (19.2%)	124 (22.6%)	
	Poor	141 (13.2%)	117 (21.4%)	
Smoke	Every day	88 (8.2%)	98 (17.9%)	<0.001
	Former	307 (28.7%)	170 (31.0%)	
	Never	638 (59.7%)	261 (47.6%)	
	Some day	36 (3.4%)	19 (3.5%)	
Region	Midwest	254 (23.8%)	121 (22.1%)	0.375
	Northeast	166 (15.5%)	73 (13.3%)	
	South	367 (34.3%)	209 (38.1%)	
	West	282 (26.4%)	145 (26.5%)	
Health	Excellent	123 (11.5%)	19 (3.5%)	<0.001
	Fair	190 (17.8%)	165 (30.1%)	
	Good	341 (31.9%)	147 (26.8%)	
	Poor	79 (7.4%)	123 (22.4%)	
	Very good	336 (31.4%)	94 (17.2%)	
Mental	No	878 (82.1%)	366 (66.8%)	<0.001
	Yes	191 (17.9%)	182 (33.2%)	
Disability	No	857 (80.2%)	309 (56.4%)	<0.001
	Yes	212 (19.8%)	239 (43.6%)	
BMI	Healthy weight	345 (32.3%)	145 (26.5%)	0.004
	Obese	365 (34.1%)	233 (42.5%)	
	Overweight	340 (31.8%)	156 (28.5%)	
	Underweight	19 (1.8%)	14 (2.6%)	
Heart attack	No	1,006 (94.1%)	498 (90.9%)	0.021
	Yes	63 (5.9%)	50 (9.1%)	
Prediabetes	No	858 (80.3%)	390 (71.2%)	<0.001
	Yes	211 (19.7%)	158 (28.8%)	
Cancer	No	834 (78.0%)	432 (78.8%)	0.755
	Yes	235 (22.0%)	116 (21.2%)	
Hypertension	No	595 (55.7%)	256 (46.7%)	0.001
	Yes	474 (44.3%)	292 (53.3%)	
High cholesterol	No	632 (59.1%)	304 (55.5%)	0.176
	Yes	437 (40.9%)	244 (44.5%)	
Arthritis	No	661 (61.8%)	258 (47.1%)	<0.001
	Yes	408 (38.2%)	290 (52.9%)	
Coronary heart disease	No	961 (89.9%)	462 (84.3%)	0.001
	Yes	108 (10.1%)	86 (15.7%)	
Stroke	No	1,001 (93.6%)	486 (88.7%)	0.001
	Yes	68 (6.4%)	62 (11.3%)	
Health insurance	No	1,003 (93.8%)	521 (95.1%)	0.365
	Yes	66 (6.2%)	27 (4.9%)	
Asthma	No	856 (80.1%)	384 (70.1%)	<0.001
	Yes	213 (19.9%)	164 (29.9%)	
Duration of dysphagia	≥180	187 (17.5%)	142 (25.9%)	<0.001
	1–6	488 (45.7%)	183 (33.4%)	
	14–29	112 (10.5%)	58 (10.6%)	
	30–89	83 (7.8%)	51 (9.3%)	
	7–13	160 (15.0%)	82 (15.0%)	
	90–179	39 (3.6%)	32 (5.8%)	

Categorical variables are presented as *n* (%). Chi-square test was used to compare differences between insomnia and non-insomnia groups. BMI, Body Mass Index.

**Table 2:** Baseline characteristics of participants with dysphonia by insomnia status.

Variables	Level	Non-insomnia (*n* = 2,286)	Insomnia (*n* = 844)	*p*
Age	≥65	760 (33.2%)	279 (33.1%)	0.042
	18–29	310 (13.6%)	117 (13.9%)	
	30–39	414 (18.1%)	134 (15.9%)	
	40–49	314 (13.7%)	96 (11.4%)	
	50–64	488 (21.3%)	218 (25.8%)	
Gender	Female	1,408 (61.6%)	552 (65.4%)	0.056
	Male	878 (38.4%)	292 (34.6%)	
Race	Hispanic (all other groups)	127 (5.6%)	42 (5.0%)	0.562
	Hispanic (Mexican/Mexican American)	157 (6.9%)	51 (6.0%)	
	Not hispanic	2,002 (87.6%)	751 (89.0%)	
Education	High school grad	482 (21.1%)	212 (25.1%)	<0.001
	Less than high school grad	146 (6.4%)	92 (10.9%)	
	More than high school grad	1,658 (72.5%)	540 (64.0%)	
Marital status	Living with partner	153 (6.7%)	74 (8.8%)	<0.001
	Married	1,017 (44.5%)	282 (33.4%)	
	Neither	1,116 (48.8%)	488 (57.8%)	
Employment	No	1,015 (44.4%)	495 (58.6%)	<0.001
	Yes	1,271 (55.6%)	349 (41.4%)	
Income	Middle and high income	1,640 (71.7%)	499 (59.1%)	<0.001
	Nearly poor	404 (17.7%)	204 (24.2%)	
	Poor	242 (10.6%)	141 (16.7%)	
Smoke	Every day	199 (8.7%)	119 (14.1%)	<0.001
	Former	607 (26.6%)	266 (31.5%)	
	Never	1,426 (62.4%)	428 (50.7%)	
	Some day	54 (2.4%)	31 (3.7%)	
Region	Midwest	549 (24.0%)	199 (23.6%)	0.243
	Northeast	334 (14.6%)	113 (13.4%)	
	South	797 (34.9%)	326 (38.6%)	
	West	606 (26.5%)	206 (24.4%)	
Health	Excellent	366 (16.0%)	61 (7.2%)	<0.001
	Fair	313 (13.7%)	227 (26.9%)	
	Good	692 (30.3%)	248 (29.4%)	
	Poor	120 (5.2%)	135 (16.0%)	
	Very good	795 (34.8%)	173 (20.5%)	
Mental	No	1,877 (82.1%)	585 (69.3%)	<0.001
	Yes	409 (17.9%)	259 (30.7%)	
Disability	No	1,973 (86.3%)	552 (65.4%)	<0.001
	Yes	313 (13.7%)	292 (34.6%)	
BMI	Healthy weight	703 (30.8%)	215 (25.5%)	0.001
	Obese	795 (34.8%)	357 (42.3%)	
	Overweight	745 (32.6%)	255 (30.2%)	
	Underweight	43 (1.9%)	17 (2.0%)	
Heart attack	No	2,186 (95.6%)	773 (91.6%)	<0.001
	Yes	100 (4.4%)	71 (8.4%)	
Prediabetes	No	1,832 (80.1%)	637 (75.5%)	0.005
	Yes	454 (19.9%)	207 (24.5%)	
Cancer	No	1,917 (83.9%)	700 (82.9%)	0.574
	Yes	369 (16.1%)	144 (17.1%)	
Hypertension	No	1,402 (61.3%)	430 (50.9%)	<0.001
	Yes	884 (38.7%)	414 (49.1%)	
High cholesterol	No	1,466 (64.1%)	503 (59.6%)	0.022
	Yes	820 (35.9%)	341 (40.4%)	
Arthritis	No	1,561 (68.3%)	443 (52.5%)	<0.001
	Yes	725 (31.7%)	401 (47.5%)	
Coronary heart disease	No	2,123 (92.9%)	742 (87.9%)	<0.001
	Yes	163 (7.1%)	102 (12.1%)	
Stroke	No	2,157 (94.4%)	776 (91.9%)	0.017
	Yes	129 (5.6%)	68 (8.1%)	
Health insurance	No	2,145 (93.8%)	802 (95.0%)	0.24
	Yes	141 (6.2%)	42 (5.0%)	
Asthma	No	1,800 (78.7%)	595 (70.5%)	<0.001
	Yes	486 (21.3%)	249 (29.5%)	
Duration of dysphonia	≥180	253 (11.1%)	170 (20.1%)	<0.001
	1–6	1,240 (54.2%)	332 (39.3%)	
	14–29	218 (9.5%)	77 (9.1%)	
	30–89	135 (5.9%)	78 (9.2%)	
	7–13	365 (16.0%)	149 (17.7%)	
	90–179	75 (3.3%)	38 (4.5%)	

Categorical variables are presented as *n* (%). Chi-square test was used to compare differences between insomnia and non-insomnia groups. BMI, Body Mass Index.

### 3.2 Risk association analysis and risk stratification analysis

Three weighted multivariate logistic regression models with varying covariate adjustments were constructed to assess the associations among dysphagia duration, dysphonia duration, and insomnia. The results showed that in all three models, the *p*-values of dysphagia and dysphonia durations were less than 0.05, and the OR values exceeded 1, indicating that dysphagia (Table 3) and dysphonia durations (Table 4) are risk factors for insomnia, and other covariates did not markedly interfere with this influence. Specifically, in Table 3, the OR value of Model 1 is 2.02 (95% CI = 1.54–2.67), *p* = 5.7 × 10^−7^, the OR value of Model 2 is 1.98 (95% CI = 1.49–2.63), *p* = 2.97 × 10^−6^, and the OR value of Model 3 is 1.41 (95% CI = 1.02–1.94), *p* = 3.63 × 10^−2^. In Table 4, the OR value of Model 1 is 1.52 (95% CI = 1.21–1.91), *p* = 2.51 × 10^−4^, the OR value of Model 2 is 1.52 (95% CI = 1.21–1.9), *p* = 3.04 × 10^−4^, and the OR value of Model 3 is 1.4 (95% CI = 1.1–1.77), *p* = 6.62 × 10^−3^.

**Table 3:** Risk association analysis between dysphagia duration and insomnia.

Exposure	Model 1 OR (95% CI)	Model 2 OR (95% CI)	Model 3 OR (95% CI)
Duration of dysphagia	2.02 (1.54–2.67)	1.98 (1.49–2.63)	1.41 (1.02–1.94)
*p*-value	5.7e−07	2.97e−06	3.63e−02

**Table 4:** Risk association analysis between dysphonia duration and insomnia.

Exposure	Model 1 OR (95% CI)	Model 2 OR (95% CI)	Model 3 OR (95% CI)
Duration of dysphonia	1.52 (1.21–1.91)	1.52 (1.21–1.9)	1.4 (1.1–1.77)
*p*-value	2.51e−04	3.04e−04	6.62e−03

**Figure 3:**
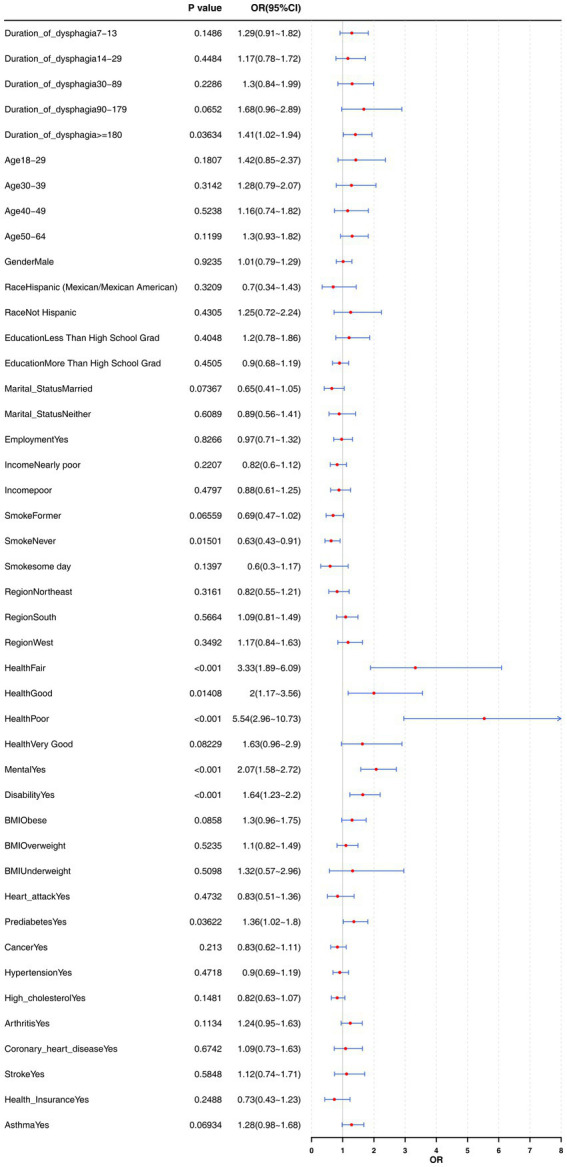
Stratified analysis of insomnia risk by dysphagia duration.

To verify the correlation stability between dysphagia duration, dysphonia duration, and insomnia risk among different populations, different groupings were made based on each covariate, and a risk stratification analysis was performed in combination with Model 3. When dysphagia duration reached or exceeded 180 days, its association with insomnia was significantly correlated and became an important risk factor for insomnia (*p* = 0.03634, OR (95% CI) = 1.41 (1.02–1.94)). In addition, five covariates were significantly correlated with insomnia, among which the protective factor was Smoke (Never) (*p* = 0.01501, OR (95% CI) = 0.63 (0.43–0.91)), and the risk factors were Health (Fair) (*p* < 0.001, OR (95% CI) = 3.33 (1.89–6.09)), Health (Good) (*p* = 0.01408, OR (95% CI) = 2 (1.17–3.56)), Health (Poor) (*p* < 0.001, OR (95% CI) = 5.54 (2.96–10.73)), Mental (Yes) (*p* < 0.001, OR (95% CI) = 2.07 (1.58–2.72)), Disability (Yes) (*p* < 0.001, OR (95% CI) = 1.64 (1.23–2.2)), and Prediabetes (Yes) (*p* = 0.03622, OR (95% CI) = 1.36 (1.02–1.8)) (Figure 3).

**Figure 4:**
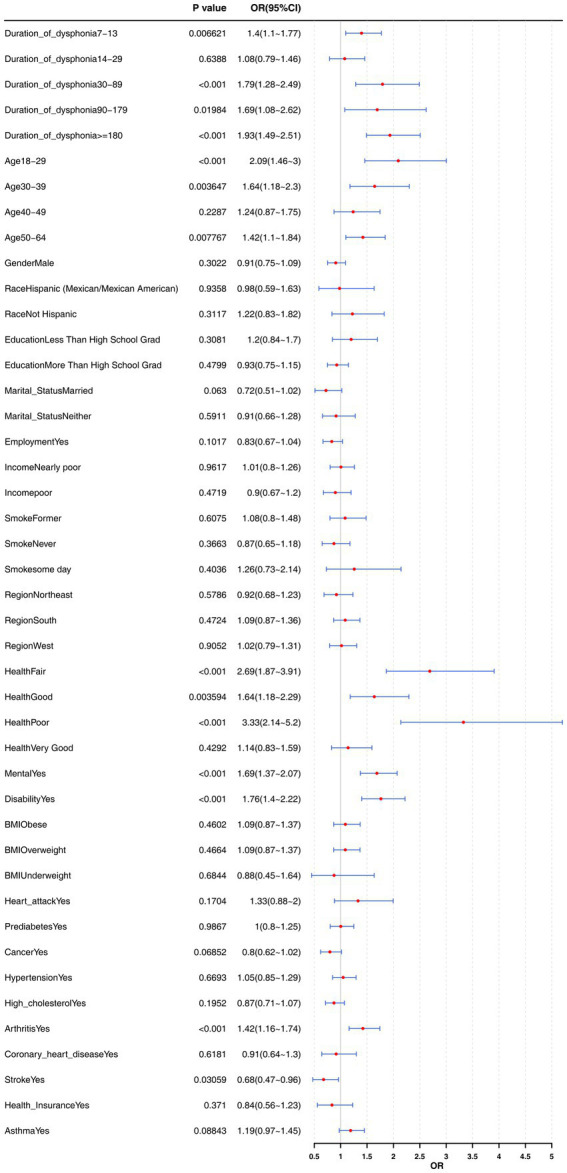
Stratified analysis of insomnia risk by dysphonia duration.

Similarly, when the dysphonia duration was 7–13 days and more than 30 days, it also showed a significant positive correlation with insomnia and became an important risk factor for insomnia (*p* < 0.05, OR > 1). In addition, among the covariates, Age (18–29, 30–39, 50–64), Health (Fair, Good, Poor), Mental (Yes), Disability (Yes), and Arthritis (Yes) were risk factors for insomnia (*p* < 0.01, OR > 1), and Stroke (Yes) was a protective factor for insomnia (*p* < 0.05, OR < 1) (Figure 4). These results provide a reference for developing intervention strategies for insomnia.

In repeated 10-fold cross-validation, Model 3 for dysphagia demonstrated moderate and stable discrimination (mean AUC = 0.696, SD = 0.041, Table 5). The direction of association for dysphagia duration was highly consistent across the resampling iterations (sign consistency: 0.98–1.00). The ≥180 days group remained statistically significant, with a median *p*-value of 0.043, indicating robust association stability (Supplementary Table S1). Other duration categories showed less consistent statistical significance (Supplementary Figure S1).

**Table 5:** Performance of Model 3 for dysphagia under repeated 10-fold cross-validation.

Mean AUC	SD AUC	Mean accuracy	SD accuracy
0.6961224	0.041302	0.2972292	0.0264927

Similarly, Model 3 for dysphonia showed stable discrimination performance (mean AUC = 0.698, SD = 0.033, Table 6). The associations between dysphonia duration and the outcome were highly consistent across the resampling iterations (sign consistency: 0.92–1.00). Several duration categories remained statistically significant, particularly the ≥180 days group, which was significant in all iterations (Supplementary Table S2). Variable importance rankings were also stable across resampling (Supplementary Figure S1).

**Table 6:** Performance of Model 3 for dysphonia under repeated 10-fold cross-validation.

Mean AUC	SD AUC	Mean accuracy	SD accuracy
0.6978489	0.0325714	0.2543785	0.0160255

### 3.3 Ranking of feature importance

**Figure 5:**
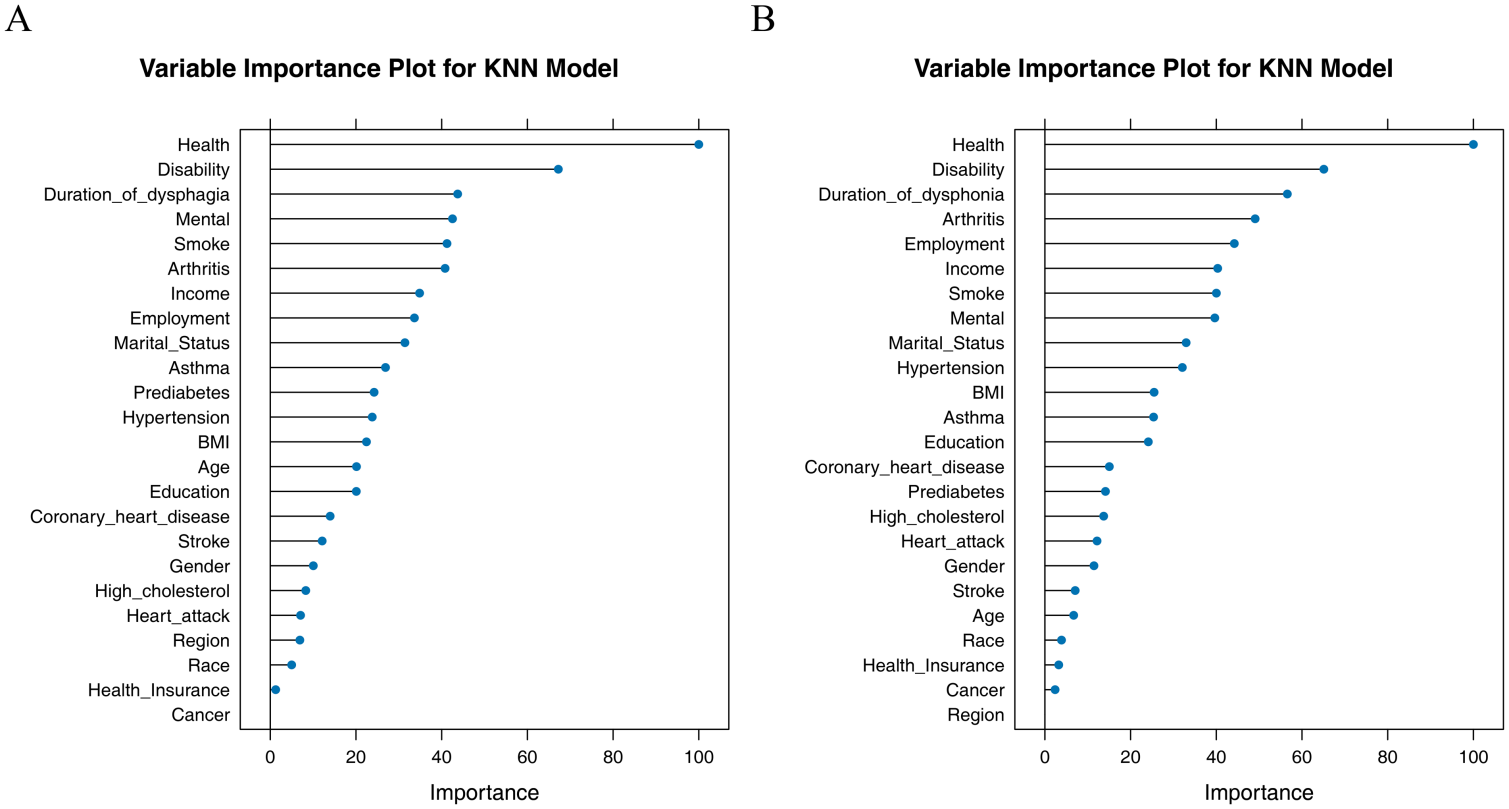
KNN analysis of the association between symptom duration and insomnia **(A,B)**. Dysphagia duration **(A)** and dysphonia duration **(B)**.

To explore the contribution of each feature to the model and its impact on insomnia, we analyzed the features in the optimal model. The results showed that dysphagia duration ranked among the top in the ranking of feature importance (Figure 5A), suggesting dysphagia duration as an important risk factor for insomnia. Likewise, in the dysphonia duration–insomnia analysis, dysphonia duration showed relatively high feature importance, suggesting a strong association with insomnia (Figure 5B). These findings highlight the importance of considering swallowing and voice disorders in insomnia prevention strategies.

## 4 Discussion

Insomnia is a prevalent sleep disorder characterized by poor sleep quality, difficulty initiating or maintaining sleep, and significant impairment in daytime functioning (39). Dysphagia is typically classified into OD and ED (19). However, voice disorders are communication impairments manifested as any difficulty in sound production that prevents the voice from fulfilling its essential role in conveying verbal and emotional information (40). Previous studies have suggested a potential link between dysphagia, speech difficulties, and insomnia. For instance, a community-based rehabilitation program involving stroke patients with dysphagia reported greater improvements in sleep among participants in the intervention group (41). Moreover, postoperative dysphagia following peroral endoscopic myotomy has been identified as a key determinant of persistent sleep disturbances in patients with achalasia (42). Additionally, comparisons between women with speech disorders and control groups have revealed differences in pain across specific head and neck regions in relation to sleep quality and insomnia indicators (43). Although the pathophysiological backgrounds of previous studies differ from those of the general population, the functional impairment of swallowing itself shares common pathways in disrupting sleep. These include nocturnal aspiration risk, physical discomfort, and associated psychological distress, which are universal stressors regardless of the primary diagnosis. Using data from the NHIS database, this study explored the association between dysphagia and dysphonia duration and insomnia occurrence. The results demonstrated that both factors were significantly correlated with insomnia when examined as independent exposures. Even after adjusting for multiple covariates, these relationships remain robust, indicating that prolonged dysphagia and speech difficulties may act as potential risk factors for insomnia. This provides important insights into its prevention and clinical management.

Results from the baseline characteristic and risk stratification analyses indicated that duration of both disorders significantly affected insomnia (*p* < 0.05). In addition, several other potential factors—age, educational attainment, marital and employment status, mental health, degree of disability, BMI, as well as medical histories of heart disease, prediabetes, hypertension, coronary artery disease, stroke, and asthma—all showed significant associations with insomnia outcomes (*p* < 0.05). A study on older adults in Japan further revealed that variables such as age, number of treated diseases, dysphagia risk assessment (DRACE) score, Geriatric Depression Scale-15 (GDS-15-J) score, and the frequency and depth of meaningful conversations were linked to poorer sleep quality (44). Notably, sleep disturbances and psychiatric disorders share a bidirectional pathogenic relationship—mental illnesses can precipitate sleep problems, while chronic insomnia itself serves as a significant precipitating factor for multiple psychiatric conditions (45). Among elderly populations, female sex, dementia, depression, anxiety, chronic pain disorders, and atrial fibrillation have all been associated with a higher prevalence of insomnia; specifically, the presence of depression, anxiety, and chronic pain substantially increases the likelihood of developing insomnia (46). Moreover, evidence suggests that nocturnal asthma symptoms correlate with poorer sleep quality, as reflected by lower mean oxygen saturation (47). Insomnia has also been identified as an independent causal risk factor for acute myocardial infarction, with a predisposition toward insomnia directly contributing to a higher risk of such cardiac events (48). Collectively, the present findings—consistent with previous research—underscore that insomnia is a multifactorial condition shaped by the interplay of demographic characteristics, physical illnesses, psychological and psychiatric factors, and specific symptoms such as dysphagia and speech impairments.

This study confirmed that dysphagia duration serves as an independent risk factor for insomnia, a finding corroborating previous research on the relationship between swallowing disorders and sleep disturbances (26). Dysphagia, beyond being an uncomfortable physiological symptom, may signal underlying health issues and is frequently associated with negative emotional states such as anxiety, depression, and social isolation (i.e., loneliness). As reported, older adults experiencing dysphagia and loneliness have a 3.476-fold higher risk of poor sleep quality compared to those without either condition, with the two factors showing additive and interactive effects among elderly individuals residing in nursing homes (49). These findings underscore the need to account for the psychological and social burdens associated with dysphagia when assessing its impact on sleep. Notably, this study’s conclusions align with findings from interventional research, including a community-based group rehabilitation program in patients with post-stroke dysphagia, suggesting that swallowing dysfunction interventions improve sleep quality (41). This indirectly suggests that the effective management of dysphagia itself can serve as a nonpharmacological approach to improving sleep quality. Furthermore, susceptibility to insomnia appears to vary across populations. One study found that individuals at risk of dysphagia were likelier to experience poor sleep quality compared to those without such a risk, and the association was particularly pronounced in men (25). Collectively, this study establishes, across a broad population, a clear independent association between dysphagia duration and insomnia occurrence, providing empirical support for understanding the underlying mechanisms linking the two. Therefore, in the clinical evaluation and prevention of insomnia, screening for swallowing function and its long-term management should be considered integral components of a comprehensive intervention strategy.

Our findings’ generalizability is bolstered by NHIS’s nationally representative design and diverse cohort. Despite geographic data limitations, our weighted multivariate model (Model 3), adjusted for extensive sociodemographic and clinical covariates, ensured robust associations. This study addresses a critical literature gap by identifying dysphagia and dysphonia as independent insomnia risk factors, regardless of age or comorbidity. Practically, integrating swallowing and vocal screenings into clinical evaluations offers a novel, nonpharmacological pathway to manage insomnia and reduce its global burden.

The importance ranking analysis revealed that factors such as dysphagia duration, dysphonia duration, overall health status, and degree of disability ranked among the top predictors, suggesting that they can serve as key indicators of insomnia risk. This observation corroborated previous findings. For instance, a study on patients with spasmodic dysphonia reported that they experienced a significantly higher burden of depression, olfactory and gustatory impairments, and non-motor sleep symptoms compared with controls (50). Our results are also consistent with research on neurological health, which demonstrated that individuals with high-level blast-induced concussions were twice as likely to report sleep disturbances compared with those with impact-related concussions—underscoring the substantial influence of neurological injury or dysfunction on sleep quality (51). Overall, these findings highlight the need for a holistic approach to insomnia prevention and intervention—one that not only emphasizes long-term management of specific symptoms such as dysphagia and dysphonia but also comprehensively evaluates an individual’s overall neurophysiological health and functional integrity. Based on the NHIS database, this study investigated the relationships among dysphagia duration, dysphonia duration, and insomnia. The results showed that dysphagia and dysphonia durations were not only significantly associated with insomnia when considered individually as exposure factors but were also found to be risk factors for insomnia after adjusting for different covariates. These findings supplement and extend the existing epidemiological evidence on risk factors for insomnia and highlight that oropharyngeal and laryngeal functional impairments may represent underrecognized contributors to sleep health. Notably, the temporal discrepancy between the assessment of dysphagia/dysphonia (past 12 months) and insomnia (past 2 weeks) aligns with a ‘chronic exposure leading to recent outcome’ framework. Since these functional impairments are typically chronic or subacute—as reflected in our analysis where symptoms lasting ≥180 days showed the strongest associations—this design captures the cumulative physiopsychological burden of long-term conditions on current sleep quality. Furthermore, focusing on the past 2 weeks for insomnia minimizes long-term recall bias and provides a more accurate snapshot of the participants’ current health status.

However, this study has certain limitations. First, insomnia was assessed via self-reported frequency rather than clinical criteria (e.g., DSM-5) or polysomnography. Although not a clinical diagnosis, these validated items are standard surrogates in large-scale epidemiological research. Additionally, owing to the NHIS database constraints, some potentially relevant variables—such as geographic region and ethnicity—could not be included in the analysis, which may have limited the models’ ability to fully capture all risk factors for insomnia. Therefore, further validation using data from diverse regions and populations is warranted to strengthen and generalize these findings.

## 5 Conclusion

Future research and clinical practice should prioritize patients with prolonged dysphagia and dysphonia durations, particularly regarding their risk of developing insomnia and the implementation of more effective treatment and intervention strategies. Such efforts may help improve patients’ quality of life and mitigate the adverse impacts of these conditions on individuals and society.

## Data Availability

The original contributions presented in the study are included in the article/Supplementary material, further inquiries can be directed to the corresponding authors.

## Footnotes

1
https://www.cdc.gov/nchs/about/index.html
