# Supplementary figures and images for: Association between duration of dysphagia and dysphonia with insomnia: results from the National Health Interview Survey
Source: Front Neurol. 2026 May 8;17:1796030. doi: 10.3389/fneur.2026.1796030 (PMC13194046; doi:10.3389/fneur.2026.1796030)

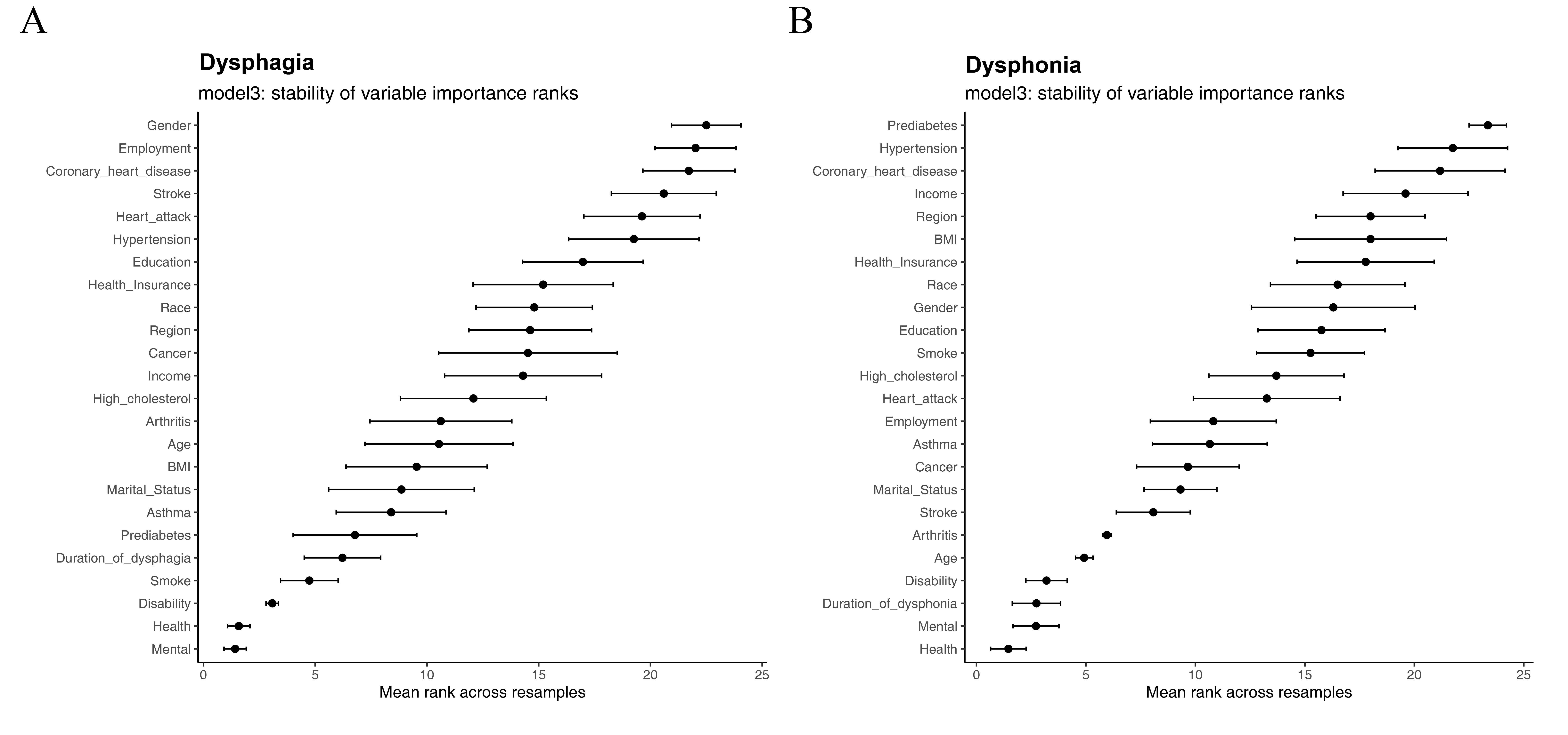

Supplement: Supplementary file 1 [file Supplementary_file_1.tif]
