# Supplementary material for: Association between duration of dysphagia and dysphonia with insomnia: results from the National Health Interview Survey
Source: Front Neurol. 2026 May 8;17:1796030. doi: 10.3389/fneur.2026.1796030 (PMC13194046; doi:10.3389/fneur.2026.1796030)
Supplement: Supplementary file 2 [file Supplementary_file_2.docx]

**Supplementary Figure S1. Stability of variable importance rankings in Model 3 under repeated 10-fold cross-validation. (A)** Dysphagia model**. (B)** Dysphonia model. Points represent the mean rank of each variable across all iterations, and error bars indicate variability (mean ± SD). Lower mean ranks indicate higher importance, while shorter error bars reflect greater stability across data splits.
